# On the distinguishability of HRF models in fMRI

**DOI:** 10.3389/fncom.2015.00054

**Published:** 2015-05-19

**Authors:** Paulo N. Rosa, Patricia Figueiredo, Carlos J. Silvestre

**Affiliations:** ^1^Flight Systems Business Unit, Aerospace, Defense & Systems Department, Deimos Engenharia, Lda.Lisboa, Portugal; ^2^Institute for Systems and Robotics and Department of Bioengineering, Instituto Superior Técnico, Universidade de LisboaPortugal; ^3^Department of Electrical and Computer Engineering, Faculty of Science and Technology, University of MacauTaipa, Macau, China

**Keywords:** HRF, fMRI, BOLD fMRI, distinguishability, model selection, experimental paradigm

## Abstract

Modeling the Hemodynamic Response Function (HRF) is a critical step in fMRI studies of brain activity, and it is often desirable to estimate HRF parameters with physiological interpretability. A biophysically informed model of the HRF can be described by a non-linear time-invariant dynamic system. However, the identification of this dynamic system may leave much uncertainty on the exact values of the parameters. Moreover, the high noise levels in the data may hinder the model estimation task. In this context, the estimation of the HRF may be seen as a problem of model falsification or invalidation, where we are interested in distinguishing among a set of eligible models of dynamic systems. Here, we propose a systematic tool to determine the distinguishability among a set of physiologically plausible HRF models. The concept of absolutely input-distinguishable systems is introduced and applied to a biophysically informed HRF model, by exploiting the structure of the underlying non-linear dynamic system. A strategy to model uncertainty in the input time-delay and magnitude is developed and its impact on the distinguishability of two physiologically plausible HRF models is assessed, in terms of the maximum noise amplitude above which it is not possible to guarantee the falsification of one model in relation to another. Finally, a methodology is proposed for the choice of the input sequence, or experimental paradigm, that maximizes the distinguishability of the HRF models under investigation. The proposed approach may be used to evaluate the performance of HRF model estimation techniques from fMRI data.

## Introduction

The hemodynamic response function (HRF) describes the local changes in cerebral blood flow, volume, and oxygenation associated with neuronal activity, and it is extensively used to model Blood Oxygen Level Dependent (BOLD) signals measured using functional Magnetic Resonance Imaging (fMRI) (Logothetis and Wandell, 2004). In general, fMRI experiments are used to map networks of brain activity that are associated with a specific stimulus or task, or that are functionally correlated during rest. Mapping of stimulus/task-related BOLD changes is most frequently achieved by fitting a general linear model (GLM) to the data, consisting on the stimulus/task time course convolved with a pre-specified HRF model (Friston et al., 1994), assuming a linear time invariant system (Boynton et al., 1996). Although the exact mechanisms underlying the HRF are not yet completely known, the consistency of its observed shape allowed for canonical (parameterized) HRF models to be derived (Friston et al., 1998). In particular, double-gamma HRF models are commonly employed in fMRI analysis. Nevertheless, extensive HRF variability has been reported across brain regions (Handwerker et al., 2004), scanning sessions (Aguirre et al., 1998), tasks (Cohen and Ugurbil, 2002), physiological modulations (Liu et al., 2004), subjects (Handwerker et al., 2004), and populations (D'Esposito et al., 2003), which may hinder or confound the measurement of BOLD changes associated with brain activity, limiting the interpretability of fMRI studies.

Common approaches attempting to take into account HRF variability allow for greater flexibility in the HRF shape and dynamics by describing it through a set of basis functions in a GLM framework. They include using the partial derivatives with respect to time and dispersion of a canonical HRF (Friston et al., 1998), finite impulse response (FIR) basis sets (Glover, 1999), and specially designed basis functions (Woolrich et al., 2004). An approach that also takes into account the spatial localization of the HRF was very recently proposed in Vincent et al. (2014). While a small number of basis functions cannot accurately model the whole range of HRF shapes and delays, at the other extreme, deconvolution of the BOLD response is a very noisy process. Critically, these approaches do not provide a biophysical foundation for the HRF model, hence limiting the physiological interpretability of the associated parameters. Moreover, they do not explain empirically observed non-linearities in the BOLD responses (Birn et al., 2001).

Biophysically informed non-linear models of the HRF have been proposed, based on the combination of the Balloon model, describing the dynamic changes in deoxyhemoglobin content as a function of blood oxygenation and blood volume (Buxton et al., 1998), with a model of the blood flow dynamics during brain activation, where neuronal activity is approximated by the stimulus/task input scaled by a factor called neural efficiency (Friston et al., 2000). In the original work that proposed this model, the associated parameters were estimated by using a Volterra kernel expansion to characterize the system dynamics (Friston et al., 2000). Later, a Bayesian estimation framework was introduced, allowing for the use of a priori distributions of the parameter values and the production of the respective posterior probability distributions given the data by using Expectation-Maximization methods (Friston, 2002). This HRF model and respective estimation procedure have further been incorporated in Dynamic Causal Models (DCM) developed to study effective connectivity among networks of brain regions from fMRI data (Friston et al., 2003). More recently, the methods of dynamic expectation maximization, variational filtering, and generalized filtering have also been proposed for model inversion (estimation) in this context (Friston et al., 2008).

Several extensions of the Balloon model have since been considered (Buxton et al., 2004), as well as a metabolic/hemodynamic model that takes the metabolic dynamics into account in order to incorporate the separate roles played by excitatory and inhibitory neuronal activities in the generation of the BOLD signal (Sotero and Trujillo-Barreto, 2007). A few alternative approaches for the estimation of these HRF models and related extensions have also been proposed (Riera et al., 2004). In Riera et al. (2004), a fully stochastic model was presented in order to include physiological noise in the hemodynamic states, in addition to the measurement noise in the observations. A local linearization filter was used for estimating the hemodynamic states as well as the model parameters. In Sotero et al. (2009), a similar approach was used for estimating the metabolic/hemodynamic model proposed by the same group. In contrast to these linearization-based approaches, Johnston et al. (2008) used particle filters so as to truly accommodate the model non-linearities. More recently, Havlicek et al. (2011) proposed non-linear cubature Kalman filtering as a means to invert models of coupled dynamical systems, which furnishes posterior estimates of both the hidden states and the parameters of the system, including any unknown exogenous input.

In fMRI experiments, the system input is given by the stimulus/task time course, which is generally designed as a series of events alternating with baseline periods at specified inter-stimulus intervals (ISIs). A number of studies have addressed the problem of systematically assessing the quality of fMRI experimental designs, both in terms of the ability to detect stimulus/task-related BOLD activation (detection power) and the ability to estimate the HRF model (estimation efficiency) in a given amount of imaging time (Dale, 1999; Liu et al., 2001). Different methodologies have been proposed to determine the optimal design of fMRI experiments for maximal estimation efficiency (Buracas and Boynton, 2002; Wager and Nichols, 2003; Maus et al., 2012), and a few studies have compared different HRF models and the associated estimation efficiency, focusing on specific parameters of interest such as the response latency and duration (Lindquist and Wager, 2007; Lindquist et al., 2009). Importantly, the authors were concerned with the physiological plausibility of the estimated HRF parameters and with their independence, such that differences in one parameter are not confounded with differences in another parameter. However, these studies were based on parameterized HRF models with no direct biophysical groundings, which severely limited the desired physiological interpretability. To our knowledge, no study has so far investigated the effect of experimental design on the estimation of biophysically informed models of the HRF.

When the HRF model is expressed as a dynamic system, the identifiability of this system must be established in order to guarantee that the HRF models inferred from the input/output data are physiologically plausible. It has been shown that the sensitivity of the HRF system input/output behavior to the model parameters is in general small, which means that, when many parameters are estimated together, their values can be varied over a large range with only small changes in the system output (Deneux and Faugeras, 2006). In these cases, the problem of model estimation may be treated as a model falsification (or invalidation) problem, in which we are interested in distinguishing among a set of eligible dynamic systems (Silvestre et al., 2010a). The simplest model falsification problem one can think of is that of stating whether or not a given model is compatible with the current observed input/output data. However, it is important to notice that a model can never be validated in practice. Indeed, the model being compatible with the input/output data up to time *t* does not imply that it should be compatible at time *t* + δ where δ > 0. Therefore, one can only say that a given model is not falsified (or invalidated) by the current input/output data. On the other hand, a model is obviously invalidated or falsified once it is not compatible with the observations. Hence, we usually refer to model falsification rather than model validation, since the latter is not achievable in practice. The related problem of model (in)distinguishability arises in a wide range of decision architectures, especially in those that are used in noisy and/or uncertain environments, where more than a single eligible model is compatible with the observed input/output dataset. The distinguishability of two models is in general affected by the input signals, particularly by the uncertainty on the input time-delay and on its magnitude. In fact, model invalidation requires a kind of persistence of the excitation condition in the exogenous inputs, so that the magnitude of the system output signal is large enough when compared to the noise level of the data acquisition process—see (Grewal and Glover, 1976; Walter et al., 1984) and references therein.

In this paper, we extend the results in Silvestre et al. (2010b), by first introducing the concept of absolutely input-distinguishable systems and showing that, for systems with forced responses, the distinguishability between two models can be significantly affected by the shape and magnitude of the external input signals. Moreover, several types of uncertainty, such as unknown input time-delays and uncertain magnitudes of the input signal, can also be adverse to model invalidation. We then exploit the concept of absolutely input-distinguishable systems, in order to optimize the estimation efficiency of fMRI experimental designs through the maximization of the distinguishability among a set of physiologically plausible HRF models. It is stressed that one of the main motivations for the work described herein is the development of a technique that helps define an optimal sequence of stimuli, so that the differences between the models in the set of plausible HRFs become apparent. Hence, the methodology proposed in this paper provides a first step to the so-called experimental paradigm design, while also shedding light on the intrinsic limitations of HRF parameter estimation based on fMRI.

## Methods

The Balloon Model proposed by Buxton et al. (1998), and further analyzed and complemented with the flow dynamics by Friston et al. (2000), consists of a non-linear differential equation that describes the dynamics of normalized values of the blood flow *b_f_*, with *s* being the vasodilatatory and activity dependent signal that increases the flow *b_f_*, the veins deoxyhemoglobin content *q*, and the blood venous volume *v*, which are considered 1 at rest. This non-linear dynamic system can be described by

(1) $$\begin{array}{l} \begin{array}{l} \begin{array}{ll} \dot{s}=\varepsilon u-k_{s}s-k_{f}(b_{f}-1) & \overset{\Delta }{=}F_{1}\\ {\dot{b}}_{f}=s & \overset{\Delta }{=}F_{2}\\ \dot{v}=\frac{1}{\tau }\left(b_{f}-v^{\frac{1}{\alpha }}\right) & \overset{\Delta }{=}F_{3}\\ \dot{q}=\frac{1}{\tau }\left(b_{f}\frac{1-{\left(1-E_{o}\right)}^{1/b_{f}}}{E_{o}}-v^{\left(\frac{1}{\alpha }-1\right)}q\right) & \overset{\Delta }{=}F_{4} \end{array}\}\overset{\Delta }{=}F(x,\theta ,u)\\ y=V_{o}\left[k_{1}\left(1-q\right)+k_{2}\left(1-\frac{q}{v}\right)+k_{3}\left(1-v\right)\right] \end{array}\}\\ \;=f(x,\theta ,u) \end{array}$$

where *x* = [*x*_1_, *x*_2_, *x*_3_, *x*_4_]^T^ = [*s*, *b_f_*, *v*, *q*]^T^, *E_o_* is the resting net oxygen extraction fraction by capillary bed, ε is the efficacy with which neuronal activity causes an increase in signal, 1/*k_s_* and 1/*k_f_* are time constants, τ is the mean transit time, and α is a stiffness exponent that specifies the flow-volume relationship of the venous balloon. The output of this model, *y*(*t*), is the BOLD signal and represents a complex response controlled by different parameters, that range from the blood oxygenation, to the cerebral blood flow, and cerebral blood volume, and reflects the regional increase in metabolism due to enhancing of the neural activity. In the output equation, *V_o_* is the resting blood volume fraction, and *k*_1_, *k*_2_, and *k*_3_ are constants.

**Figure 1:**
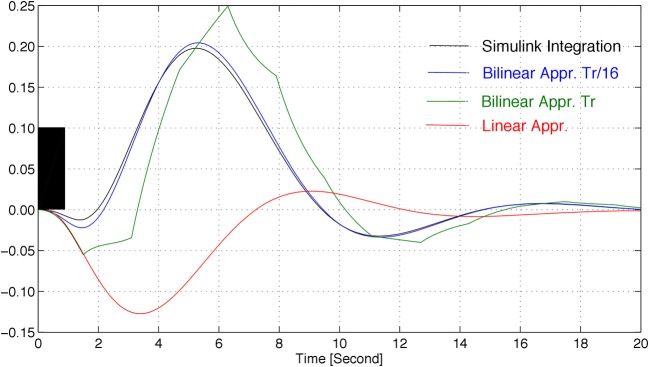
**Approximations of the response of model Equation (1) to a rectangular input signal (in black), for the parameters of Table 1, where *TR* denotes the repetition time**.

The response of the system described by Equation (1), with the parameters in Table 1 and with initial state *x*^T^ (0) = [0 1 1 1], to a rectangular input signal, is depicted in Figure 1, for different integration periods.

**Table 1:** **Parameters for the non-linear model described by Equation (1)**.

**Parameter**	**ε**	***k_s_*[s^−1^]**	***k_f_*[s^−1^]**	**τ[s]**	**α**	***E_o_***	***V_o_***	***k*_1_**	***k*_2_**	***k*_3_**
Value	0.065	0.550	0.410	1.280	0.880	0.920	4.88	7E_o_	2.0	2E_o_ −0.2

The linear approximation of the model of the system leads to pronouncedly different responses, when compared to the non-linear system. An alternative to this, as described in sequel, is to consider a so-called bilinear model, which accurately mimics the non-linear behavior for sufficiently small integration periods.

### Linearization and discretization of the model

The model described by Equation (1) is highly non-linear and parameter-dependent, thus barely allowing any systematic analysis of the associated expected behavior. Hence, to make the problem tractable from a mathematical point of view, the (bi)linearization of the HRF is considered in this paper. This approach allows the use of a widely spread framework for analysis, namely that of the linear time-varying systems. Figure 1 shows that a close match of the HRF can be obtained by using a bilinear approximation (linear on the state, if the input is fixed, and linear on the input, if the state is fixed). Therefore, in this subsection, a (bi)linearization is derived that approximates the non-linear model locally and that is able to describe the state of the system at a given time, *x*(*kT_s_*), as a function of the state several sampling periods before, *x* ((*k* − *N*)*T_s_*).

In particular, linearizing Equation (1) around *x*(·) = x^*^ and *u*(·) = 0, i.e., writing the associated Taylor expansion and truncating it at the linear term, one obtains (omitting the time-dependence of the variables, for the sake of readability):

$$\begin{array}{l} \dot{x}\approx F(x^{*},\theta ,0)+{\frac{\partial F(x,\theta ,u)}{\partial x}|}_{x^{*},\theta ,0}(x-x^{*})\\ \;+\sum_{i}u_{i}\left({\frac{\partial^{2}F(x,\theta ,u)}{\partial x\partial u_{i}}|}_{x^{*},\theta ,0}(x-x^{*})+{\frac{\partial F(x,\theta ,u)}{\partial u_{i}}|}_{x^{*},\theta ,0}\right), \end{array}$$

where

(2) $$\frac{\partial F}{\partial x}=\left[\begin{array}{cccc} -k_{s} & -k_{f} & 0 & 0\\ 1 & 0 & 0 & 0\\ 0 & \frac{1}{\tau } & -\frac{x_{3}^{\left(\frac{1}{\alpha }-1\right)}}{\alpha \tau } & 0\\ 0 & \frac{\partial F_{4}}{\partial x_{2}} & \frac{\partial F_{4}}{\partial x_{3}} & -\frac{x_{3}^{\left(\frac{1}{\alpha }-1\right)}}{\tau } \end{array}\right],$$

$$\begin{array}{l} \frac{\partial F_{4}}{x_{2}}=\frac{1}{\tau }\left[\frac{1-{\left(1-E_{o}\right)}^{\frac{1}{x_{2}}}}{E_{o}}+\frac{\log(1-E_{o}){\left(1-E_{o}\right)}^{\frac{1}{x_{2}}}}{E_{o}x_{2}}\right],\\ \frac{\partial F_{4}}{x_{3}}=\frac{1}{\tau }\left(1-\frac{1}{\alpha }\right)x_{3}^{\left(\frac{1}{\alpha }-2\right)}x_{4}. \end{array}$$

and with output equation described by

$$\frac{\partial y}{\partial x}=\left[\begin{array}{c} 0,0,-k_{3}V_{o}+k_{2}V_{o}qv^{-2},-k_{1}V_{o}-k_{2}V_{o}v^{-1} \end{array}\right].$$

Moreover, given that *F*_1_ depends linearly upon *u*, we have that $\frac{\partial^{2}F}{\partial x\partial u_{i}}=0$.

Using the transformation proposed in Friston et al. (2000), one finally obtains the following dynamics:

(3) $$\dot{\tilde{x}}=A\tilde{x}+\sum_{i}\;u_{i}\;E_{i}\tilde{x},$$

where $\tilde{x}={\left[\begin{array}{cc} 1 & x \end{array}\right]}^{\text{T}}$,

$$\begin{array}{l} A\overset{\Delta }{=}\left[\begin{array}{cc} 0 & 0\\ \left(F(x^{*},\theta ,u)-\frac{\partial F(x^{*},\theta ,u)}{\partial x}x^{*}\right) & \frac{\partial F(x^{*},\theta ,0)}{\partial x} \end{array}\right],\\ E_{i}\overset{\Delta }{=}\left[\begin{array}{cc} 0 & 0\\ \frac{\partial F(x^{*},\theta ,0)}{\partial u_{i}} & 0 \end{array}\right], \end{array}$$

and $\frac{\partial F(x^{*},\theta ,0)}{\partial u_{i}}={\left[\begin{array}{cccc} \varepsilon & 0 & 0 & 0 \end{array}\right]}^{\text{T}}.$

#### Uncertain dynamic model description

It should be noticed that the dynamics in Equation (3) are bilinear in the state and input variables. This non-linear term hinders the distinguishability analysis proposed in Rosa and Silvestre (2011) and, thus, a more suitable description is derived in herein.

For the sake of simplicity, we start by redefining $x(t)\overset{\Delta }{=}\tilde{x}(t)$ and $x^{*}(t)\overset{\Delta }{=}{\left[1\;{\left(x^{*}(t)\right)}^{\text{T}}\right]}^{\text{T}}$. It was previously shown that the continuous-time dynamic model of the HRF, for a single input, can be approximated by

(4) $$\{\begin{array}{lll} \dot{x}(t) & = & (A(t)+B_{o}(t)u(t)+\Delta (t)B_{1}(t)u(t))x(t),\\ & & \;x(0)=x^{*}(0),\\ y(t) & = & h(x(t)), \end{array}$$

with *t* ≥ 0, and where Δ :R^+^ → R was also included to represent an input uncertainty subject to |Δ(*t*)|≤ 1 for all *t* ≥ 0, and where *B_o_* = *E*_1_. This input uncertainty can be seen as a surrogate for uncertainty in the stimulation signal. The initial state is denoted by *x*(0) ∈ *R^n^*, and *n* is the number of states of the system. Moreover, we assume that

$$B_{1}(t)=\eta B_{o}(t),$$

with known η ∈ *R*. We also define *B*(*t*) = *B_o_* (*t*) + Δ(*t*)*B*_1_ (*t*).

To proceed with the derivation of a discrete-time description of the HRF model in Equation (4), for a given sampling period, *T_s_*, the following assumptions are posed:

**Assumption 1**: The input signal, *u*(·), is constant during sampling periods, i.e., *u*(*t*) = *u*(*kT_s_*), for all *t* ∈ [*kT_s_*, (*k* + 1)*T_s_*[.

**Assumption 2**: The input uncertainty, Δ (·), is constant during sampling periods, i.e., Δ (*t*) = Δ(*kT_s_*), for all *t* ∈ [*kT_s_*, (*k* + 1)*T_s_*[.

**Assumption 3**: The maps *A*(·), *B_o_* (·), and *B*_1_ (·), are constant during sampling periods, i.e., *A*(*t*) = *A*(*kT_s_*), *B_o_* (*t*) = *B_o_* (*kT_s_*), and *B*_1_ (*t*) = *B*_1_ (*kT_s_*), for all *t* ∈ [*kT_s_*, (*k* + 1)*T_s_*[.

Under these assumptions, the system in Equation (4) can be rewritten as

(5) $$\{\begin{array}{lll} \dot{x}(t) & = & \tilde{A}\left(k,\Delta (k)\right)x(t),\;x(0)=x^{*}(0),\\ y(t) & = & g(\tilde{x}(t)), \end{array}$$

for $\tilde{x}(t)\in [kT_{s},(k+1)T_{s}]$, and where

$$\tilde{A}\left(k,\Delta (k)\right)=A_{o}(k)+\Delta (kT_{s})A_{1}(k),$$

with

$$A_{o}(k)=A(kT_{s})+B_{o}(kT_{s})u(kT_{s}),$$

and

$$A_{1}(k)=B_{1}(kT_{s})u(kT_{s}).$$

In the sequel, we will abbreviate *x*(*k*) = *x*(*kT_s_*), for the sake of simplicity. We are now in conditions of stating the following proposition:

**Proposition 1:** Define

$$I^{*}=\left[\begin{array}{cccc} 0 & 0 & \cdots & 0\\ 0 & 1 & \ddots & \vdots \\ \vdots & \ddots & \ddots & 0\\ 0 & \cdots & 0 & 1 \end{array}\right],$$

and

$$\phi (k)=V(k)\Lambda^{*}(k)V^{-1}(k)e^{A(kT_{s})T_{s}}-V(k)\Lambda^{*}(k)V^{-1}(k)-I^{*},$$

where *V*(*k*) Λ (*k*)*V*^−1^ (*k*) = *A*(*kT_s_*)*T_s_* is the spectral decomposition of *A*(*kT_s_*)*T_s_* with Λ (*k*) diagonal and Λ _11_ (*k*) = 0, and

$$\Lambda_{ij}^{*}(k)=\{\begin{array}{rr} \frac{1}{\Lambda_{ij}(k)}, & \text{if i=j and }\Lambda_{\text{ij}}\neq \text{0},\\ 0, & \text{otherwise}. \end{array}$$

Furthermore, let

$$\begin{array}{l} G_{o}(k)=e^{A(k)}+B_{o}(k)u(k)+\phi (k)B_{o}(k)u(k)\text{ and}\\ G_{1}(k)=B_{1}(k)u(k)+\phi (k)B_{1}(k)u(k). \end{array}$$

Then, the system in Equation (5) is described by

(6) $$\{\begin{array}{l} x(k+1)\;=\;G\left(k,\Delta (k)\right)x(k),\;x(0)=x^{*}(0),\\ y(k)\;=\;h(x(k)), \end{array}$$

where

$$G\left(k,\Delta (k)\right)=G_{o}(k)+\Delta (k)G_{1}(k),$$

and for *x*(*k*) = *x*(*kT_s_*). *Proof:* See Appendix A in Supplementary Material.

Notice that Equation (6), with *G*(*k*, Δ (*k*)) = *G_o_* (*k*) + Δ(*k*)*G*_1_ (*k*), associated with the linearization of the output map, *g*, is a full description of the HRF dynamics by means of a linear model with known matrices, *G_o_* (*k*) and *G*_1_ (*k*), and an uncertain parameter, Δ (*k*). This description, however, is bilinear in the state, *x*(*k*), and model uncertainty, Δ (*k*). This bilinear relationship is tainted once we describe the state *x*(*k* + 1) as a function of *x*(*k* − 1). Nevertheless, notice that

(*G_o_* (*k* + 1) + Δ*G*_1_ (*k* + 1))(*G_o_* (*k*) + Δ*G*_1_ (*k*)) = *G_o_* (*k* + 1)*G_o_* (*k*) +Δ(*G*_1_ (*k* + 1)*G_o_* (*k*) + *G_o_* (*k* + 1)*G*_1_ (*k*)), since *G*_1_ (*k* + 1)*G*_1_ (*k*) = 0 and where, for the time being, we considered that Δ is constant (but unknown), i.e., Δ (*k*) = Δ for all *k*. To see this, notice that

$$\begin{array}{l} G_{1}(k+1)G_{1}(k)=(B_{1}(k+1)+\phi (k+1)B_{1}(k+1))(B_{1}(k)\\ \;+\phi (k)B_{1}(k))\\ \;=\underset{=0}{\underset{︸}{B_{1}(k+1)B_{1}(k)}}+B_{1}(k+1)\phi (k)B_{1}(k)\\ \;+\phi (k+1)\underset{=0}{\underset{︸}{B_{1}(k+1)B_{1}(k)}}+\\ \;+\phi (k+1)B_{1}(k+1)\phi (k)B_{1}(k), \end{array}$$

and that *B*_1_ (*k* + 1) ϕ (*k*)*B*_1_ (*k*) = 0, due to the fact that the first row of ϕ is zero, and that all but the first column of *B*_1_ are also zero.

By proceeding in a similar manner, we conclude that

$$\begin{array}{l} \left(G_{o}(k+m)+\Delta G_{1}(k+m)\right)\cdots (G_{o}(k)\text{​​​​​}+\text{​​​​}\Delta G_{1}(k))\\ \;=\Psi_{o}(k+m)\text{​​​​​}+\text{​​​​​}\Delta \Psi_{1}(k+m), \end{array}$$

where

$$\Psi_{o}(k+m)=G_{o}(k+m)\cdots G_{o}(k),$$

and

$$\{\begin{array}{lll} \Psi_{1}(k) & = & G_{1}(k),\\ \Psi_{1}(k+m) & = & \begin{array}{l} G_{o}(k+m)\Psi_{1}(k+m-1)\\ +G_{1}(k+m)\Psi_{0}(k+m-1). \end{array} \end{array}$$

Hence, the state *x*(*k* + *m* + 1) can be written as

$$x(k+m+1)=\left(\Psi_{o}(k+m)+\Psi_{1}(k+m)\Delta \right)x\left(k\right)$$

Furthermore, the non-linear output Equation of (1) can be linearized as

(8) $$y(x)=y(x^{*})+{\frac{\partial y}{\partial x}|}_{x^{*}}(x-x^{*}),$$

which, in turn, can alternatively written as:

(9) $$z=y(x)-y(x^{*})+{\frac{\partial y}{\partial x}|}_{x^{*}}x^{*}={\frac{\partial y}{\partial x}|}_{x^{*}}x.$$

where *z*(*t*) can be seen as the measurement for the linear time-varying system obtained by the linearization of Equation (1).

### Absolutely distinguishable systems

The problem of indistinguishability typically arises from large amplitudes of the measurement noise, small intensity of the input excitation signals, model uncertainty, and uncertain initial conditions. In particular, if the Signal-to-Noise Ratio (SNR) of the measurements is not sufficiently large, one may be able to explain the observed variables by using more than a single dynamic model, from the set of *eligible* models. A similar conclusion applies if the intensity of the input signal is not sufficient to excite the dynamics of the system.

This section will therefore propose a methodology to systematically derive conditions that guarantee the distinguishability of a set of dynamic models, regardless of the noise sequences and initial states.

#### Systems with uncertain initial state

We start by analyzing the case where the dynamics of the system are known, although the initial state is uncertain and the measured variables are corrupted by bounded noise. Using Equation (8), we have that

(10) $$y(k)=\underset{\bar{y}(k)}{\underset{︸}{y(x^{*}(k))-C(k)x^{*}(k)}}+C(k)x(k)+n(k),$$

where

$$C(k)={\frac{\partial y}{\partial x}|}_{x^{*}(k)},$$

and where *n*(*k*) is the measurement noise. Consider that a given input sequence, *u*(0), …, *u*(*N*), feeds the inputs of systems *S_A_* and *S_B_*, respectively described by

$$S_{A}:\{\begin{array}{lll} x_{A}(k+1) & = & G_{A}(k)x_{A}(k),\\ y_{A}(k) & = & {\bar{y}}_{A}(k)+C_{A}(k)x_{A}(k)+n_{A}(k), \end{array}$$

$$S_{B}:\{\begin{array}{lll} x_{B}(k+1) & = & G_{B}(k)x_{B}(k),\\ y_{B}(k) & = & {\bar{y}}_{B}(k)+C_{B}(k)x_{B}(k)+n_{B}(k), \end{array}$$

where *y_A_* and *y_B_* are defined as in Equation (10), and $|n_{A}(k)|\leq \frac{\bar{n}}{2}$, $|n_{B}(k)|\leq \frac{\bar{n}}{2}$. Moreover, we assume that *x_A_* (0) ∈ *X*_o_ and *x_B_* (0)∈ X_o_, where *X_o_* ∈ *R^n^* is a convex polytope. Let ϕ_*i*_ = [*n^T^_i_*, *u^T^_i_*]^T^, denote the measurement noise, *n_i_* ∈ *W* ⊆ *R^n^_n_*, and input signals, *u_i_* ∈ *U* ⊆ *R^n^_u_*, at time instant *i*.

**Definition 1**: Systems *S_A_* and *S_B_* are said *absolutely (*X*_0_, *U*, *W*)-input distinguishable in *N* sampling times* if, for any non-zero

$$\left(x_{A}(0),x_{B}(0),\phi_{1},\phi_{2},\cdots ,\phi_{N}\right)\in X_{o}\times X_{o}\times \overset{N\text{times}}{\overset{︷}{\Phi \times \cdots \times \Phi }},$$

where ϕ_*i*_ ∈ *W* × *U* =:Φ ⊆ *R^n^_u_* + *n_d_* for *i* = 0, 1, …, *N*, there exists *k* ∈ {0, 1, …, *N*} such that

$$y_{A}(k)\neq y_{B}(k).$$

Moreover, two systems are said *absolutely (*X_o_*, *U*, *W*)-input distinguishable* if there exists *N* ≥ 0 such that they are absolutely (*X_o_*, *U*, *W*)-input distinguishable in *N* sampling times.

Let *U* = (*u*(0), *u*(1), …, *u*(*N*)) and

$$W=\left\{\left(n(0),n(1),\cdots ,n(N)\right):\underset{0\leq k\leq N}{\forall }|n(k)|\leq \frac{\bar{n}}{2}\right\}.$$

The following proposition can be used to state whether a pair of systems is distinguishable or not.

**Proposition 2**: Systems *S_A_* and *S_B_* are absolutely (*X_o_*, *U*, *W*)-input distinguishable in *N* sampling times if and only if a solution to the following linear problem does not exist:

(11) $$\begin{array}{l} \left[\begin{array}{cc} C_{A}(0) & -C_{B}(0)\\ -C_{A}(0) & C_{B}(0)\\ C_{A}(1)G_{A}(0) & -C_{B}(0)G_{B}(0)\\ -C_{A}(1)G_{A}(0) & C_{B}(0)G_{B}(0)\\ \vdots & \vdots \\ C_{A}(N)G_{A}(N-1)\cdots G_{A}(0) & -C_{B}(0)G_{B}(N-1)\cdots G_{B}(0)\\ -C_{A}(N)G_{A}(N-1)\cdots G_{A}(0) & C_{B}(0)G_{B}(N-1)\cdots G_{B}(0)\\ M_{o} & 0\\ 0 & M_{o} \end{array}\right]\\ \text{ ​​​​​​}\left[\begin{array}{c} x_{A}(0)\\ x_{B}(0) \end{array}\right]\leq \left[\begin{array}{c} \bar{n}-{\bar{y}}_{A}(0)+{\bar{y}}_{B}(0)\\ \bar{n}+{\bar{y}}_{A}(0)-{\bar{y}}_{B}(0)\\ \bar{n}-{\bar{y}}_{A}(1)+{\bar{y}}_{B}(1)\\ \bar{n}+{\bar{y}}_{A}(1)-{\bar{y}}_{B}(1)\\ \vdots \\ \bar{n}-{\bar{y}}_{A}(N)+{\bar{y}}_{B}(N)\\ \bar{n}+{\bar{y}}_{A}(N)-{\bar{y}}_{B}(N)\\ m_{o}\\ m_{o} \end{array}\right], \end{array}$$

where *X_o_* is defined so that *x* ∈ *X_o_* ⇔ *M_o_ x* ≤ *m_o_*, which can be written as *X_o_* = Set(*M_o_*, *m_o_*).

*Proof:* See Appendix B in Supplementary Material.

#### Systems with uncertain model

We now consider the case where the system dynamics are uncertain and described by

$$\begin{array}{l} S_{A}:\{\begin{array}{ccc} x_{A}(k+1) & = & \left(G_{o}^{A}(k)+\Delta_{A}G_{1}^{A}(k)\right)x_{A}(k),\\ y_{A}(k) & = & {\bar{y}}_{A}(k)+C_{A}(k)x_{A}(k)+n_{A}(k), \end{array}\\ S_{B}:\{\begin{array}{ccc} x_{B}(k+1) & = & \left(G_{o}^{B}(k)+\Delta_{B}G_{1}^{B}(k)\right)x_{B}(k),\\ y_{B}(k) & = & {\bar{y}}_{B}(k)+C_{B}(k)x_{B}(k)+n_{B}(k), \end{array}\;\; \end{array}$$

where *y_A_* and *y_B_* are defined as in Equation (10), and $|n_{A}(k)|\leq \frac{\bar{n}}{2}$, $|n_{B}(k)|\leq \frac{\bar{n}}{2}$. We also assume that |Δ_*A*_ | ≤ 1 and |Δ_*B*_ | ≤ 1. Moreover, for this case we assume that *X_o_* is a singleton, thus removing the uncertainty in the initial state. In this case, *S_A_* and *S_B_* denote families of systems, due to the uncertainties Δ_*A*_ and Δ_*B*_. Therefore, the introduction of the following definition is required.

**Definition 2**: The families of systems *S_A_* and *S_B_* are said absolutely (*X_o_*, *U*, *W*)-input distinguishable in *N* sampling times if, for any pair of realizations (*S*_1_, *S*_2_) ∈ *S_A_* × *S_B_*, the systems *S*_1_ and *S*_2_ are absolutely (*X_o_*, *U*, *W*)-input distinguishable in *N* sampling times.

Hence, we are now in condition to state the following proposition:

**Proposition 3**: The families of systems *S_A_* and *S_B_* are absolutely (*X_o_*, *U*, *W*)-input distinguishable in *N* sampling times if and only if there does not exist a solution to the following linear problem:

(12) $$\Theta_{N}\left[\begin{array}{c} \Delta_{A}\\ \Delta_{B} \end{array}\right]\leq \theta_{N},$$

where

$$\Theta_{N}=\left[\begin{array}{cc} 0 & 0\\ 0 & 0\\ C_{A}(1)\Psi_{1}^{A}(0)x_{A}(0) & -C_{B}(1)\Psi_{1}^{B}(0)x_{B}(0)\\ -C_{A}(1)\Psi_{1}^{A}(0)x_{A}(0) & C_{B}(1)\Psi_{1}^{B}(0)x_{B}(0)\\ \vdots & \vdots \\ C_{A}(N)\Psi_{1}^{A}(N-1)x_{A}(0) & -C_{B}(N)\Psi_{1}^{B}(N-1)x_{B}(0)\\ -C_{A}(N)\Psi_{1}^{A}(N-1)x_{A}(0) & C_{B}(N)\Psi_{1}^{B}(N-1)x_{B}(0) \end{array}\right]$$

and

$$\theta_{N}=\left[\begin{array}{c} \bar{n}-{\bar{y}}_{A}(0)+{\bar{y}}_{B}(0)-C_{A}(0)x_{A}(0)+C_{B}(0)x_{B}(0)\\ \bar{n}+{\bar{y}}_{A}(0)-{\bar{y}}_{B}(0)+C_{A}(0)x_{A}(0)-C_{B}(0)x_{B}(0)\\ \bar{n}-{\bar{y}}_{A}(1)+{\bar{y}}_{B}(1)-C_{A}(1)\Psi_{o}^{A}(0)x_{A}(0)+\Psi_{o}^{B}(0)x_{B}(0)\\ \bar{n}+{\bar{y}}_{A}(1)-{\bar{y}}_{B}(1)+C_{A}(1)\Psi_{o}^{A}(0)x_{A}(0)-\Psi_{o}^{B}(0)x_{B}(0)\\ \vdots \\ \bar{n}-{\bar{y}}_{A}(N)+{\bar{y}}_{B}(N)-C_{A}(N)\Psi_{o}^{A}(N-1)x_{A}(0)+\Psi_{o}^{B}(N-1)x_{B}(0)\\ \bar{n}+{\bar{y}}_{A}(N)-{\bar{y}}_{B}(N)+C_{A}(N)\Psi_{o}^{A}(N-1)x_{A}(0)-\Psi_{o}^{B}(N-1)x_{B}(0) \end{array}\right]$$

*Proof:* See Appendix C in Supplementary Material.

**Figure 2:**
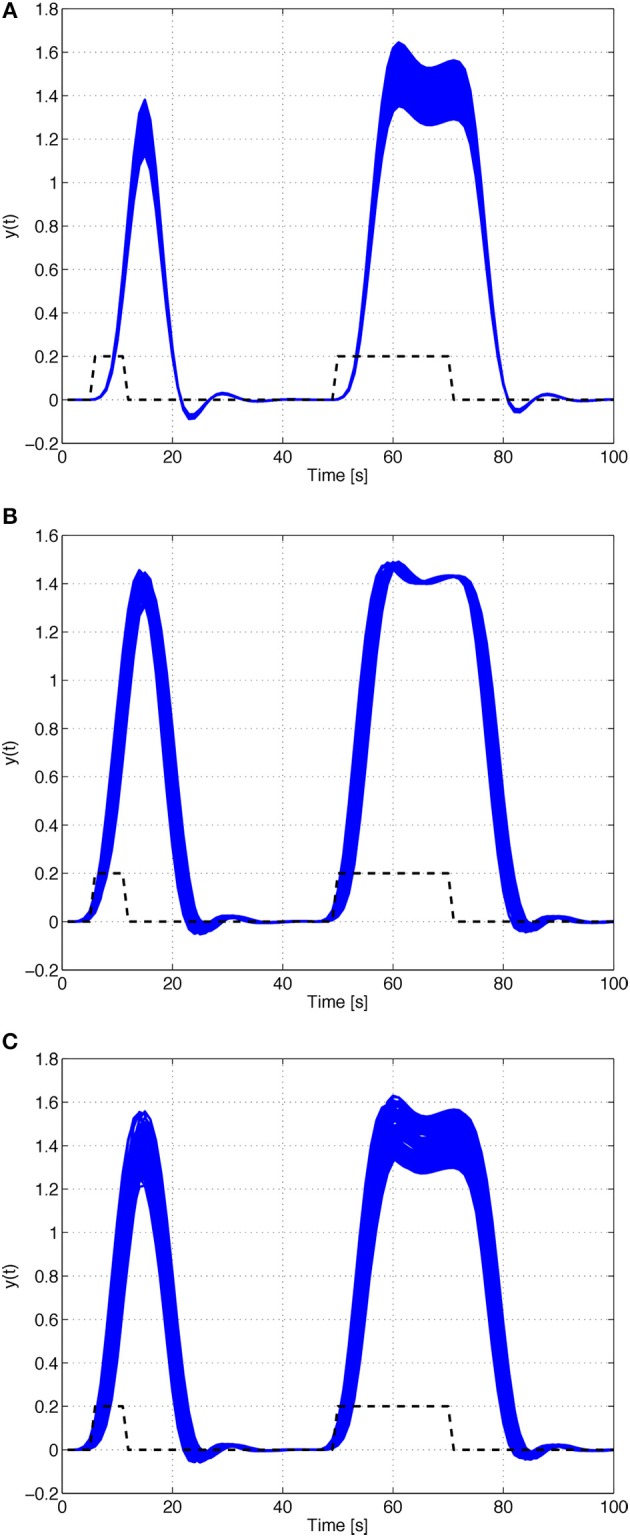
**Impulse and step responses of the HRF model with the parameters of Table 1. (A)** 10% uncertainty on the input signal. **(B)** Uncertain input time-delay. **(C)** Uncertain model and input time-delay.

Figure 2A depicts the impulse and step responses of the HRF model with the parameters of Table 1, with an uncertainty of 10% in the input signal. It should be noticed that this type of uncertainty mainly affects the amplitude of the responses of the system. Thus, the rise- and fall-times are not significantly influenced by small variations on the amplitude of the input signal.

#### Systems with uncertain input time-delays

In this subsection, a strategy to model uncertain input time-delays is developed. The approach presented in the sequel amounts for rewriting these uncertain input time-delays as model uncertainty.

Consider that the input signal, at sampling time *k*, is given by

$$u(k)=\tilde{u}(k-k_{d}),$$

where *k_d_* is an integer (the uncertain delay) satisfying |*k_d_*| ≤ *k*_*d*_, with known *k*_*d*_. The value of *u*(*k*), for each *k* ≥ 0, is also assumed known and bounded. Thus, we have, for each *k* ≥ 0,

(13) $$\underline{u}(k)\leq u(k)\leq \bar{u}(k),$$

where *u*(*k*) = max_|*m*|≤*k*_d__ *u*(*k* − *m*) and *u*(*k*) = min_|*m*|≤*k*_*d*__ *u*(*k* − *m*). Therefore, Equation (13) can be rewritten as

$$u(k)=u_{o}(k)+\Delta_{u}(k)u_{1}(k),$$

where $|\Delta_{u}(k)|\leq 1,u_{o}(k)=\frac{\bar{u}(k)+\underline{u}(k)}{2}$, and $u_{1}(k)=\frac{\bar{u}(k)-\underline{u}(k)}{2}$.

Hence, unknown but bounded time-delays on the input can be treated as uncertainty on the *B* matrix. The impulse and step responses of the HRF model with the parameters of Table 1, with an uncertain input time-delay, *k_d_*, bounded by |*k_d_*| ≤ 3, are depicted in Figure 2B. As seen in the figure, the uncertainty in the input time-delay enlarges the uncertainty in the rise- and fall-times of the output.

#### Systems with uncertain model and input time-delays

For the sake of completeness, in this subsection we analyze the effects of simultaneous uncertainty on the model and on the input time-delays. The results for this scenario are depicted in Figure 2C. As expected, the uncertainty on the model chiefly affects the amplitude of the responses, while the uncertainty on the input time-delay changes the corresponding rise- and fall-times.

#### Systems with uncertain model and uncertain initial state

We now consider the case where both the system dynamics and the initial state are uncertain. The problem is set to that of concluding whether the following two families of systems are distinguishable:

$$\begin{array}{l} S_{A}:\{\begin{array}{ccc} x_{A}(k+1) & = & (G_{o}^{A}(k)+\Delta_{A}(k)G_{1}^{A}(k))x_{A}(k),\\ y_{A}(k) & = & {\bar{y}}_{A}(k)+C_{A}(k)x_{A}(k)+n_{A}(k), \end{array}\\ S_{B}:\{\begin{array}{ccc} x_{B}(k+1) & = & (G_{o}^{B}(k)+\Delta_{B}(k)G_{1}^{B}(k))x_{B}(k),\\ y_{B}(k) & = & {\bar{y}}_{B}(k)+C_{B}(k)x_{B}(k)+n_{B}(k), \end{array}\;\; \end{array}$$

where *y_A_* and *y_B_* are defined as in Equation (10), and $|n_{A}(k)|\leq \frac{\bar{n}}{2}$, $|n_{B}(k)|\leq \frac{\bar{n}}{2}$. We also assume that |Δ_*A*_ (*k*)| ≤ 1 and |Δ_*B*_ (*k*) ≤ 1. Moreover, for this case we assume that *X_o_* is a convex polytope.

**Proposition 4:** Let *e*_1_ = [ 1 0 0 0 0]^T^. The families of systems *S_A_* and *S_B_* are absolutely (*X_o_*, *U*, *W*)-input distinguishable in *N* sampling times if and only if there does not exist a solution to the following linear problem: 
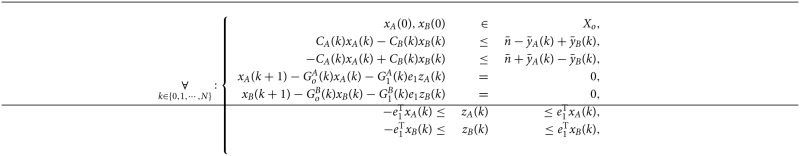
 where the unknown variables are *x_A_* (0), … *x_A_* (*N*), *x_B_* (0), … *x_B_* (*N*), *z_A_* (0), … *z_A_* (*N* − 1), and *z_B_* (0), … *z_B_* (*N* − 1).

*Proof:* See Appendix D in Supplementary Material.

**Figure 3:**
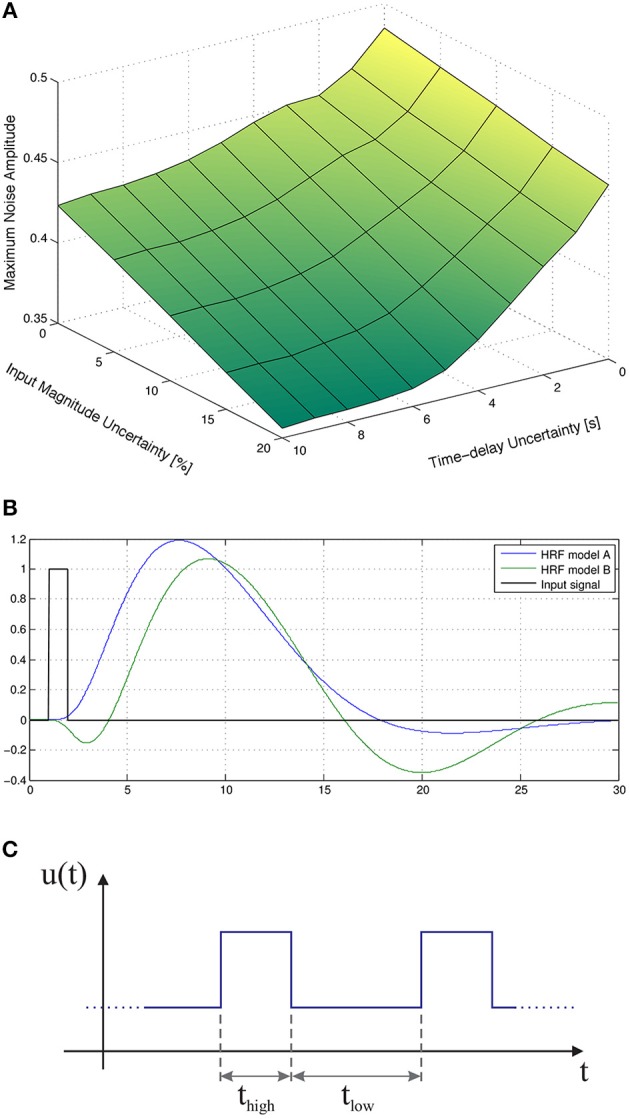
**Maximum amplitude of the measurement noise that guarantees the absolute distinguishability of two particular families of models. (A)** As a function of the uncertainty on the input signal and on the corresponding time-delay. **(B)** As a function of the uncertainty on the magnitude and time-delay of the input signal, for a deterministic input signal. **(C)** As a function of the uncertainty on the magnitude and time-delay of the input signal, for a stochastic input signal with mean *t*_high_ and *t*_low_ of 12 s obtained from a uniform distribution of width 12 s.

Figure 3A depicts the maximum amplitude of the measurements noise that guarantees the absolute distinguishability of two particular families of HRF models, as a function of the uncertainty on the input signal and on the corresponding time-delay. As expected, the maximum level of sensor noise such that the two families of models are absolutely distinguishable, decreases with both types of uncertainty.

### Pre-processing of fMRI time series

We stress that the assumption that the additive noise in the measured signal is bounded is not conservative in practice, since outliers and other *unboundedness* behaviors can, in general, be tackled during pre-processing, i.e., before performing the main analysis of the signals. This can be done, in particular, by low-pass filtering the signal, so that high-frequency noise is significantly attenuated.

Additionally, the following pre-processing steps are commonly applied to fMRI time series data before submitting them to statistical analysis (Jezzard et al., 2001): (i) normalization of the whole 4D fMRI dataset by scaling each volume by a single (common) scaling factor, so that subsequent analyses are valid; (ii) motion correction by alignment of all fMRI volumes to a reference volume in the time series, usually performed by applying rigid-body transformations, in order to reduce the effect of subject head motion during the experiment; and (iii) high-pass temporal filtering, usually using a local fit of a straight line (Gaussian-weighted within the line to give a smooth response), in order to remove low-frequency artifacts such as signal drifts or physiological fluctuations.

## Results

In this section, we study the influence of the choice of the input signal on the distinguishability of a set of HRF models. A methodology to optimize the fMRI experimental design that takes advantage of this knowledge is also presented.

Throughout the remainder of this paper, we are going to refer to the families of HRF models *A* and *B*, described by the dynamics in Equation (1), with the physiologically plausible parameters presented in Table 2. Model family *B* displays a pronounced undershoot and the presence of an initial dip, in stark contrast to model family *A*.

**Table 2:** **Parameters for the families of non-linear models**.

**HRF**	***k_s_* [s^−1^ ]**	***k_f_* [s^−1^ ]**	**τ [s]**	**α**	***E_o_***
*A*	0.400	0.100	2.080	0.320	0.340
*B*	0.220	0.110	2.180	0.320	0.985

**Figure 4:**
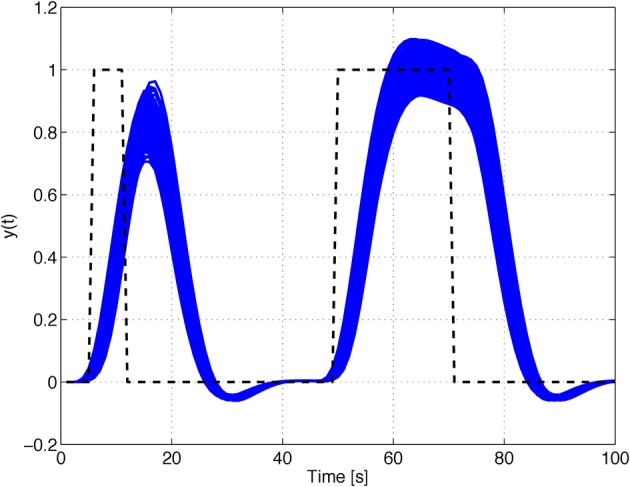
**Time responses of the nominal models of families *A* and *B***.

The response of the nominal HRF models, for the parameter configurations of Table 2, with initial state *x*^T^ (0) = [0 1 1 1], to a rectangular input signal of duration 1 s and unit magnitude, is depicted in Figure 4.

**Figure 5:**
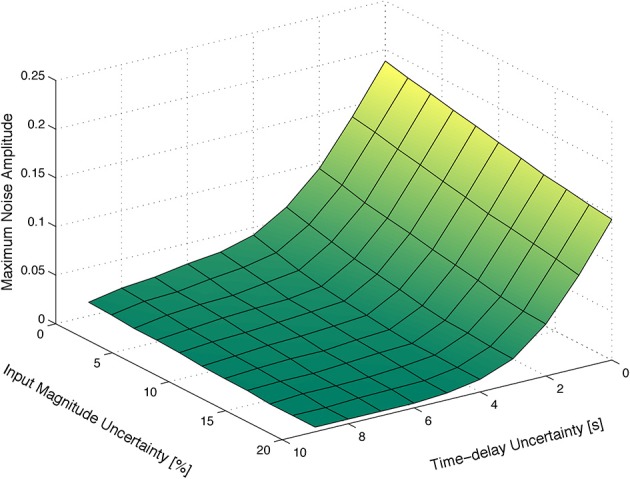
**Input signal adjustable parameters**.

In general, the input signal is composed of a series of rectangular pulses (events) of duration *t*_high_ alternating with baseline periods of duration *t*_low_, with a total duration of 200 s (see Figure 5).

**Figure 6:**
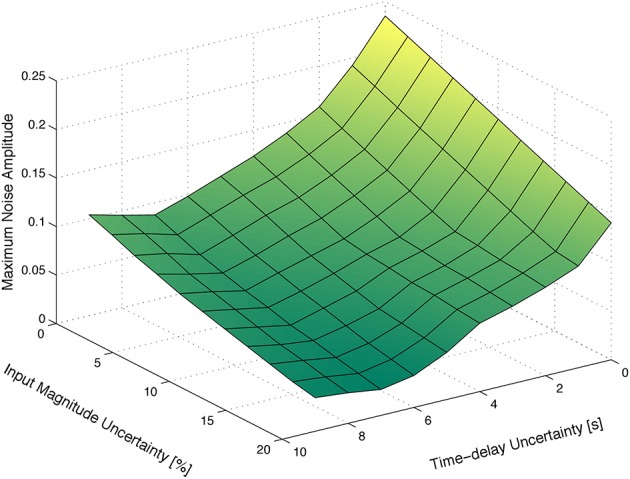
**Rectangular input responses of family *A* with uncertain input magnitude and input time-delay**.

In order to illustrate the characteristic behavior of HRF model family *A*, their responses to rectangular input signals of duration 5 and 20 s and unit magnitude, with an uncertain input time-delay, *k_d_*, bounded by |*k_d_*| ≤ 3 s, and input uncertainty of 10%, are depicted in Figure 6. The uncertainty on the input time-delay enlarges the uncertainty in the rise- and fall-times of the output, while the uncertainty in the input mainly affects the amplitude of the responses of the system.

Figure 3B depicts the maximum amplitude of the measurements noise that guarantees the absolute distinguishability of the families of models *A* and *B*, for an input signal with *t*_low_ = 12 s and *t*_high_ = 12 s, as a function of the uncertainty on the magnitude of the input signal and on the corresponding time-delay. As expected, the maximum level of measurement noise such that the families of models *A* and *B* are absolutely distinguishable decreases with both types of uncertainty.

Furthermore, we considered a stochastic input signal, composed of a series of rectangular pulses with mean duration of *E*(*t*_high_) = 12 s, and mean baseline period of *E*(*t*_low_) = 12 s drawn from a uniform distribution of width 12 s. According to the results in the literature (see, for instance, Josephs et al., 1997; Miezin et al., 2000), we observe that, by performing random small variations on *t*_high_ and *t*_low_, alternative trajectories of the non-linear model Equation (1) are exploited, which in turn improves the identifiability of the models, as depicted in Figure 3C.

We now analyze the effect of different experimental designs on the distinguishability of the families of models at hand. At this point, our goal is to find the combination of values of *t*_low_ and *t*_high_ such that the absolute distinguishability of two or more families of models is guaranteed for the highest upper bound on the amplitude of the measurement noise. We denote this optimal combination of values by (t^*^_low_, t^*^_high_). The advantage of using an input signal with parameters (t^*^_low_, t^*^_high_) obviously stems from the fact that we can allow for the highest amplitude on the measurement noise, while guaranteeing the distinguishability of the families.

**Figure 7:**
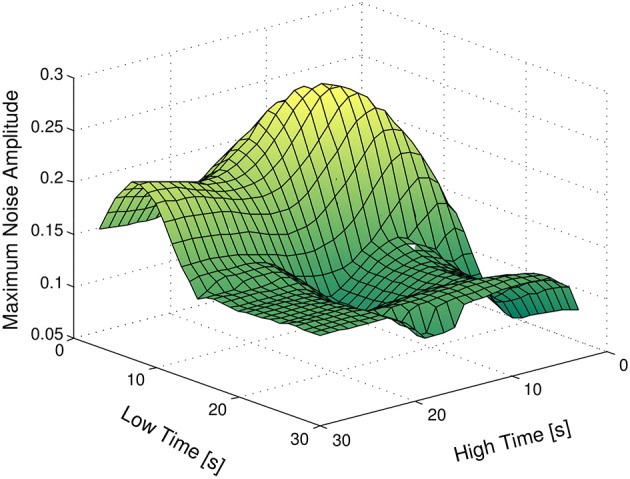
**Maximum amplitude of the measurement noise guaranteeing the absolute distinguishability of the families *A* and *B*, as a function of *t*_low_ and *t*_high_**.

Figure 7 depicts the results obtained, considering no time-delay or magnitude uncertainty. As expected, input signals with very small values of *t*_high_ and large values of *t*_low_ do not have the power required to significantly stimulate the system. On the other hand, input signals with very small values of *t*_high_ and *t*_low_ are faster than the dynamics of the system, and hence do not produce remarkable changes in the output of the plant. As a final remark, the optimum value for *t*_low_ and *t*_high_ is 10 s, i.e., t^*^_low_ = 10 s and t^*^_high_ = 10 s.

## Discussion

We have addressed the problem of the distinguishability of HRF models in the analysis of fMRI data of brain activation, based on the biophysically informed description of the HRF as a non-linear time-invariant input-state-output dynamic system. We first introduced the concept of absolutely input-distinguishable systems and then showed that the distinguishability between two HRF models, and hence system identification, is significantly affected by the external input (stimulus/task) signals. In particular, the uncertainty in the input time-delays and its magnitude may adversely affect model identification, by reducing the maximum noise level below which model distinguishability is guaranteed. We then applied the concept of absolutely input-distinguishable systems to the development of a methodology for the assessment of the HRF estimation efficiency of fMRI experimental designs, through the maximization of the distinguishability level among a set of physiologically plausible HRF models.

The main contribution of this paper is therefore 2-fold. On the one hand, we show that the distinguishability of two HRF models depends on the level of the measurement noise as well as on the characteristics of the input signal. On the other hand, we develop a methodology to optimize fMRI experimental designs for HRF estimation, which maximizes the allowable noise amplitude that does not impair the distinguishability of a set of a priori admissible dynamic systems.

In this paper, it is assumed that the system inputs can be selected or, at least, measured. This assumption is verified in a straightforward manner when external inputs are present, such as sensory stimuli or cognitive tasks. Although no explicit external inputs exist in resting-state fMRI acquisitions, it has been observed that discrete neuronal events do occur (Deco and Jirsa, 2012). Most interestingly, it has been recently suggested that such events can be identified as peaks of relatively large BOLD signal amplitude (Tagliazucchi et al., 2011), and resting-state fMRI data can then be seen as “spontaneous event-related” data (Wu et al., 2013).

### Significance of HRF estimation

The importance of estimating the HRF in fMRI experiments is based on the extensively observed variability of its shape and dynamics across brain regions, conditions, subjects, and populations, with critical consequences in the analysis of fMRI data. In fact, one direct consequence of HRF variability is that the deviation of the real HRF from the pre-specified HRF leads to a poorer model of the observed BOLD signal and hence reduces the sensitivity to detect BOLD changes (Handwerker et al., 2004). Another consequence is the potential detection of a group effect due to a systematic HRF difference, which would then be incorrectly interpreted as a neuronal effect. Moreover, when attempting to infer causality within brain networks from BOLD data, differences in HRF latency across brain regions can potentially confound the directionality of information flow (David et al., 2008; Smith et al., 2011; Murta et al., 2012; Jorge et al., 2014). On the other hand, HRF variability may be an object of interest on its own, potentially reflecting physiological changes associated with the effects of drugs, aging or pathology, for example (Iadecola, 2004). Additionally, there is a growing interest in studying, not only the amplitude of BOLD activation, but also its dynamics, namely its latency and duration, which are reflected in the HRF (Bellgowan et al., 2003). In these cases, it would be desirable to estimate the actual HRF model underlying the BOLD signal measured in each voxel, experiment, subject or population, or otherwise account for its variability.

Despite the acknowledged need for modeling the HRF underlying fMRI BOLD data, and although different approaches have been continuously proposed in the literature for this purpose, our ability to understand HRF variability remains poor (Handwerker et al., 2012). Critically, most studies have focused on parameterized HRF models in a linear framework, while the estimation of physiologically plausible non-linear HRF models with direct biophysical interpretability has been very limited. In particular, no previous study has investigated the optimal fMRI experimental design for the estimation of such biophysical HRF models. We believe that our work therefore makes an important contribution for understanding how a biophysically informed model of the HRF may be inferred from fMRI data, as a function of experimental design and measurement noise.

### Biophysically informed HRF modeling

Using a biophysically informed model of the HRF not only allows for a physiologically plausible interpretation of the results, but it also more accurately explains empirical BOLD data, particularly concerning commonly observed non-linearities. Importantly, in contrast to parameterized HRF models, biophysical models described by dynamic systems can account for the detailed dynamics of BOLD responses through a reduced number of parameters, while constraining it to be physiologically plausible. For example, the post-stimulus undershoot and the initial dip are two features of observed BOLD responses that naturally emerge from this dynamic system under slightly different combinations of a limited number of parameters. Although using such dynamic systems represents an additional computational effort compared to the more straightforward linear methods, this may nevertheless become the chosen approach in studies where a detailed characterization of the BOLD temporal dynamics is desirable. In particular, the combination of EEG with fMRI may greatly benefit from such approaches (Riera et al., 2005). On the other hand, important complementary information may be gained for HRF model estimation by combining BOLD recordings with the acquisition of blood flow data using Arterial Spin Labeling (ASL) or near-infrared spectroscopy (NIRS) (Huppert et al., 2006). Despite the potential advantages of such a biophysically informed dynamic system approach to fMRI data analysis, only a few studies have been dedicated to the associated problem of system identification/model estimation (Friston, 2002; Riera et al., 2004). Our study therefore makes a significant contribution to this limited body of literature, by introducing the concept of input-distinguishability of HRF models in order to inform model selection in this context.

### Optimization of the experimental design

Previous studies systematically assessing the quality of fMRI experimental designs have again been focused on parameterized HRF models within a linear framework (Dale, 1999; Liu et al., 2001). They found that optimal estimation efficiency is obtained at the cost of reduced detection power by employing randomized rapid event-related designs. In fact, it was shown that, if the ISI is properly jittered or randomized from trial to trial, the efficiency improves monotonically with decreasing mean ISI (Dale, 1999). In general, it is found that a trade-off exists between detection power and estimation efficiency, with block designs being optimal for the former while event-related designs are optimal for the latter (Liu et al., 2001). Nevertheless, a recent report established the feasibility and test-retest reliability of estimating HRF parameters from block design fMRI data (Shan et al., 2014). In our work, we have used a randomized design by introducing uncertainty in the ISI, and we showed that smaller uncertainty leads to better distinguishability for the same noise level. Our results are therefore consistent with the literature.

## Limitations

The framework adopted in this work resorts to deterministic concepts and, therefore, certain assumptions are posed on the signals acting on the system, in particular in terms of maximum amplitudes. Stochastic approaches are more flexible in that sense, but require the knowledge regarding the statistical properties of those signals, which may not be trivial to obtain, or which may be violated in practice. Therefore, a compromise between these two alternative frameworks—deterministic and stochastic—for the distinguishability of HRF models is still a subject of further research.

## Conclusion

In summary, in this paper we proposed a novel approach to assess distinguishability among a set of physiologically plausible biophysically informed HRF models, and to design fMRI experiments for optimal estimation efficiency of such HRF models, with potentially great impact in further understanding HRF variability and its physiological meaning.

### Conflict of interest statement

The authors declare that the research was conducted in the absence of any commercial or financial relationships that could be construed as a potential conflict of interest.
