# Supplementary material for: On the distinguishability of HRF models in fMRI
Source: Front Comput Neurosci. 2015 May 19;9:54. doi: 10.3389/fncom.2015.00054 (PMC4460732; doi:10.3389/fncom.2015.00054)
Supplement: Supplementary file 1 [file DataSheet1.DOCX]

## Appendix A

*Proof of Proposition 1:* The system in (5) can be discretized by

$$\left\{\begin{matrix} x(k+1) & = & G\left(k,\Delta(k)\right)x(k), x(0)=x^{*}(0), \\ y(k) & = & h(x(k)), \end{matrix}\right.$$

where

$$G\left(k,\Delta(k)\right)=e^{\left(A_{o}(k)+\Delta(k)A_{1}(k)\right)T_{s}}.$$

Moreover, from the structural properties of $A(t)$ and $B(t)$, we have that, for all $t_{1},t_{2},t_{3}\geq0$,

$\left\{\begin{matrix} B(t_{1})B(t_{2}) & = & 0, \\ A(t_{1})B(t_{2})A(t_{3}) & = & 0, \\ B(t_{1})A(t_{2})B(t_{3}) & = & 0. \end{matrix}\right.$ (7)

Hence, using (7), we obtain, by omitting the time dependence, $t$,

$$\begin{matrix} e^{A+Bu} & = & I+A+Bu+\frac{1}{2}\left(A+Bu\right)^{2}+\frac{1}{3!}\left(A+Bu\right)^{3}+\cdots\\ & = & I+A+Bu+\frac{A^{2}+ABu+BuA}{2}+\frac{A^{3}+BuA^{2}+A^{2}Bu}{3!}+\cdots\\ & = & e^{A}+Bu+\sum_{i=1}^{+\infty} \frac{BuA^{i}+A^{i}Bu}{(i+1)!} \\ & = & \underbrace{e^{A}+B_{o}u+\varphi B_{o}u}_{G_{0}}+\Delta\underbrace{\left(B_{1}+\varphi B_{1}\right)u}_{G_{1}}, \end{matrix}$$

where we also used the fact that $B_{o}\varphi=0$ and $B_{1}\varphi=0$. ◼

## Appendix B

*Proof of Proposition 2:* Systems $S_{A}$ and $S_{B}$ are not absolutely $(X_{o},U,W)$-input distinguishable in $N$ sampling times if and only if

$$|y_{A}(k)-y_{B}(k)|\leq\bar{n}, \text{for} \text{some} x_{A}(0),x_{B}(0)\in X_{o},$$

for all $k=0,1,\cdots,N$, which is equivalent to

which can be rewritten as the linear problem (11). ◼

## Appendix C

*Proof of Proposition 3:* According to Definition 2.3.1, the families of systems $S_{A}$ and $S_{B}$ are not absolutely $(X_{o},U,W)$-input distinguishable in $N$ sampling times if and only if

$$|y_{A}(k)-y_{B}(k)|\leq\bar{n}, \text{for} \text{some} x_{A}(0),x_{B}(0)\in X_{o},$$

for $k=0,1,\cdots,N$, and for some pair of realizations $(\Delta_{A},\Delta_{B})$, which is equivalent to

and, in general, for any $0\leq k\leq N$,

which can be rewritten as the linear problem (12). ◼

## Appendix D

*Proof of Proposition 4:* According to Definition 2.3.1, the families of systems $S_{A}$ and $S_{B}$ are not absolutely $(X_{o},U,W)$-input distinguishable in $N$ sampling times if and only if

$$|y_{A}(k)-y_{B}(k)|\leq\bar{n}, \text{for} \text{some} x_{A}(0),x_{B}(0)\in X_{o},$$

for $k=0,1,\cdots,N$, and for some sequence of uncertainties $\Delta_{A}(0),\cdots,\Delta_{A}(N-1)$, $\Delta_{B}(0),\cdots,\Delta_{B}(N-1)$, which is equivalent to satisfying

Notice that $G_{1}^{A}(k)$ and $G_{1}^{B}(k)$ only have non-zero elements in the first column, which means that we can write

$$\left\{\begin{matrix} G_{1}^{A}(k)x_{A}(k) & = & G_{1}^{A}(k)e_{1}e_{1}^{\text{T}}x_{A}(k), \\ G_{1}^{B}(k)x_{B}(k) & = & G_{1}^{B}(k)e_{1}e_{1}^{\text{T}}x_{B}(k). \end{matrix}\right.$$

However, since $e_{1}^{\text{T}}x_{A}(k)=1$ and $e_{1}^{\text{T}}x_{B}(k)=1$, we obtain

$$\left\{\begin{matrix} z_{A}(k) & \mathop{=}\limits^{\Delta} & \Delta_{A}(k)e_{1}^{\text{T}}x_{A}(k), \\ z_{B}(k) & \mathop{=}\limits^{\Delta} & \Delta_{B}(k)e_{1}^{\text{T}}x_{B}(k), \end{matrix}\right.$$

satisfying $|z_{A}(k)|\leq1$, $|z_{B}(k)|\leq1$.
